# Preclinical Characterization of a Novel Anti-Cancer PD-L1 Inhibitor RPH-120

**DOI:** 10.3389/fphar.2021.723038

**Published:** 2021-08-11

**Authors:** Andrey Kulikov, Elena Shipaeva, Anastasia Dmitrieva, Vera Batrak, Georgy Shipunov, Colin Guy, Jill Smith, Ran Zhang, Michael Zhang, Jeff Duan, Anton Chestukhin, Sergei Barbashov, Mikhail Samsonov, Yan Lavrovsky

**Affiliations:** ^1^ JSC R-Pharm, Moscow, Russia; ^2^ Covance Laboratories Ltd, Harrogate, United Kingdom; ^3^ PharmaLegacy, Shanghai, China; ^4^ Affinity Molecules, LLC, Richmond, VA, United States; ^5^ R-Pharm Overseas Inc, San Diego, CA, United States

**Keywords:** cancer immunotherapy, monoclonal antibody, PD-L1, *in vitro* efficacy, animal models, therapeutic agent

## Abstract

RPH-120 is a novel fully human anti-PD-L1 IgG1 monoclonal antibody with specifically designed Asn300Ala mutation in Fc fragment. Surface plasmon resonance assay showed that affinity of the RPH-120 to the dimeric form of human PD-L1-Fc fusion protein was much higher than affinity to the monomeric His-tagged PD-L1. Further binding studies demonstrated that RPH-120 is able to bind to human and monkey but not mouse PD-L1. Tissue cross-reactivity study showed good comparability of human and Cynomolgus monkeys tissue staining. Bioactivity was assessed using mixed lymphocyte reaction assay. This study revealed that RPH-120 was able to activate T cells preventing PD1/PD-L1 interaction. Antitumor efficacy was analyzed in HCC-827 lung cancer xenografts in humanized CD34^+^ mice at three dosage levels: 20, 80, and 200 mg/kg. RPH-120 demonstrated significant tumor growth inhibition, and this inhibition was comparable to that of atezolizumab. In a single dose toxicity, toxicokinetic and dose range finding study performed in Cynomolgus monkeys, RPH-120 was administered *via* intravenous (IV) bolus or 60-min IV infusion, followed by 8-weeks recovery period. An acceptable toxicokinetic profile was demonstrated and administration at doses of up to 200 mg/kg was well tolerated by all animals. In conclusion, RPH-120 revealed promising *in vitro* and *in vivo* activity and safety. RPH-120 is a potent anti-PD-L1 drug candidate for cancer immunotherapy.

## Introduction

Cancer immunotherapy is attracting increasing attention as an effective therapeutic option. The growing knowledge as to how the immune system works, in particular with regard to fighting cancer has provided the way for the rational development of novel treatment strategies (Pennock and Chow, 2015). Under physiological conditions, checkpoint mechanisms maintain tolerance to self-antigens and prevent immune system mediated damage (Keir et al., 2008). Tumors exploit the same molecular and cellular mechanisms to avoid immune detection in order to escape elimination. The immunosuppressive strategies utilized by tumor microenvironment include involvement of diverse cell types (e.g., regulatory T cells, type 2 macrophages, etc.) as well as molecular mechanisms, such as expression of co-inhibitory receptors on tumor infiltrating effector cells such as so called “immune checkpoints” (Zou and Chen, 2008; Chen and Mellman, 2013). The immune checkpoint molecules regulate the immune balance, therefore, neutralization of immunosuppressive checkpoints possibly leads to cancer elimination. Among these immune checkpoints, the programmed death 1 receptor (PD-1) is an important regulator of the maintenance of immune tolerance in healthy state, but in cancer the PD-1 system may provide cancer cells an opportunity to avoid immune response providing tolerance to malignant cells within the tumor microenvironment (Francisco et al., 2010; McDermott and Atkins, 2013). Interaction of PD-1 with its naturally occurring ligands, PD-L1 (B7–H1, CD274) and PD-L2 (B7–DC, CD273), induces an inhibitory signal resulting in reduction of T cell proliferation, cytokine production, and cytotoxic activity. PD-L1 interaction with PD-1 may be one of the mechanisms that enables tumors to evade immunesurveillance by directly limiting effector T cell activity. PD-L1 is expressed on immune cells including resting T cells, B cells, dendritic cells, natural killers (NKs), and macrophage, as well as nonhematologic cells, such as cells of placenta. Interestingly, PD-L1 expressed on antigen-presenting cells (APCs) can also bind CD80 expressed on T cell, and this binding reduces T cell activation and cytokine production (Butte et al., 2007). PD-L1 was also shown to be expressed on the surface of tumor cells of various solid malignancies (Wu et al., 2019). Overexpression of the PD-L1 in tumor is associated with a worse prognosis across numerous tumor histologies, making PD-1/PD-L1 immunomodulatory axis attractive for the therapeutic intervention (Wang et al., 2016). Therefore, treatment approaches targeting PD-1 and PD-L1 interaction aim to overcome immune resistance by restoring immunological detection and activation of effector functions.

In addition to the functional studies, efforts were made to investigate structural properties of PD-L1 and other B7 family members. Both biochemical and X-ray crystallography studies have shown that human PD-L1 exists in membrane-fixed and soluble states (Ikemizu et al., 2000), and the soluble form is always present as a monomer (Bailly and Vergoten, 2020). It was suggested that the dimerization of PD-L1 may be promoted at the interface of the interacting cells. Chen et al. proposed a model that PD-1 binds to the dimeric PD-L1, which was reasonable taking in account absence of steric hindrance (Chen et al., 2010). Thus, we hypothesized that preferential binding of therapeutic antibody to dimeric form of PD-L1 may give functional advantage in realizing anti-cancer potential.

The block of the PD-1/PD-L1 interaction by monoclonal antibodies is one of the most successful example of immunotherapies with demonstrated sustained antitumor responses in several tumor types (Wu et al., 2019). This novel approach prevents PD-1/PD-L1-induced immune evasion of tumor cells. Currently, several anti-PD-1 (pembrolizumab, nivolumab, cemiplimab) and anti-PD-L1 therapeutic antibodies (atezolizumab, durvalumab, avelumab) are widely used for treatment of various types of cancer (Jiang et al., 2019; Han et al., 2020). These commercial antibodies have shown overwhelming success for advanced cancers such as melanoma and non-small lung cancers. These antibodies demonstrated unprecedented positive outcomes in clinical trials and have changed the standards of cancer treatment. Anti-PD-1 and anti-PD-L1 inhibitors have common features but also some intrinsic and clinically relevant differences. It was hypothesized that anti-PD-L1 antibodies may be less toxic, since they do not bind PD-L2. A number of extensive systematic comparisons of PD-1 and PD-L1 inhibitors safety profiles support this statement (Xu et al., 2018; Banna et al., 2020), whereas one meta-analysis study revealed that toxicities of anti-PD-1 and anti-PD-L1 inhibitors are quite comparable (Duan et al., 2020).

Here, we describe development and characterization of the novel anti-PD-L1 agent RPH-120, a fully human anti-PD-L1 IgG1 class monoclonal antibody. RPH-120 discovery process was designed to identify antibodies that preferentially recognize dimeric form of PD-L1 and able to block PD-1/PD-L1 interaction and signaling. In most experiments RPH-120 activity was compared to that of atezolizumab because it is the best characterized anti-PD-L1 therapeutic antibody. We anticipate that RPH-120 will become a member of the therapeutic PD-L1 inhibitors with functional properties distinct from previously reported molecules.

## Materials and Methods

Antibody generation*.* Fully human antibodies against human PD-L1 were selected from an antibody library using yeast display approach. Yeast cells harboring antibodies specific for dimeric hPD-L1 were isolated from the library by binding to biotinylated form of hPD-L1-Fc fusion protein captured by streptavidin magnetic beads. Dimerization of the hPD-L1-Fc fusion protein was achieved by intrinsic dimerization of the Fc-fragment of the molecule. After several rounds of selection, screening of individual Ab-expressing clones was carried out using PD-L1-Fc dimer.

Surface plasmon resonance (SPR) analysis of RPH-120 interaction with PD-L1 was performed in two studies. In the first study binding affinity of RPH-120 was determined toward monomeric and dimeric forms of human PD-L1 and compared with those for atezolizumab. In the second binding study RPH-120 binding affinity to PD-L1 from three different species was evaluated: human, Cynomolgus monkey and mouse. The affinity was measured by kinetic titration analysis using SPR biosensor. The proteins conjugated to the CM5 chip include 1) monoclonal anti-human IgG antibodies (GE Healthcare) and 2) PD-L1-Fc fusion protein (R&D Systems). For the three species study, two forms of PD-L1 ligand, dimeric PD-L1-Fc fusion protein (R&D Systems) and monomeric His-tagged PD-L1 (R&D Systems), were used. For both forms of the ligand, RPH-120 was directly immobilized onto a CM5 sensor chip *via* amine coupling. His-tagged and Fc-tagged PD-L1 proteins from human, Cynomolgus monkey and mouse were obtained from R&D Systems and diluted to 500 nM in HBS-EP running buffer. Activation was carried out by injecting EDC/NHS activation agent into the chip at 10 µl/min for 8 min. For dimeric PD-L1-Fc ligand immobilized on chip, series of varying concentrations of RPH-120 or atezolizumab have been applied. Each PD-L1 protein was injected over immobilized RPH-120 at 30 µl/min for 1 min and the binding response was monitored. Series of sensograms were generated and analyzed using kinetic evaluation of 1:1 binding model.

For binding of monomeric His-tagged PD-L1 ligand antibody capturing approach was used. To the chip with immobilized anti-human antibodies, test antibodies, RPH-120 or atezolizumab, were loaded at 25 µl/min for 2 min. The ligand was loaded at 25 µl/min for 3 min. Series of sensograms for captured RPH-120 or atezolizumab at different PD-L1-His ligand concentrations were generated and used for analysis. The study analyzing monomeric and dimeric forms of human PD-L1 was performed using Reichert Technologies Instrument, model SR75000DC and TraceDrawer analysis software and the study evaluating PD-L1 from three different species was performed using Biacore instrument, model T200.

Tissue cross-reactivity (TCR) study was performed using immunohistochemical (IHC) techniques using cryo-sections from selected human and Cynomolgus monkey panels of tissues (36 tissue types, three donors were used). The panel of tissues included: adrenal, urinary bladder, blood cells, blood vessel, bone marrow, breast, brain, eye, fallopian tube, heart, intestine, kidney, liver, lung, lymph node, oesophagus, ovary, pancreas, parotid (salivary gland), peripheral nerve, pituitary, placenta, prostate, skin, spinal cord, spleen, stomach, striated muscle, testis, thymus, thyroid, tonsil, ureter, uterus – cervix, and uterus – endometrium. To facilitate IHC detection RPH-120 and human IgG1 were conjugated to Alkaline Phosphatase using the commercial LYNX Rapid Alkaline Phosphatase Antibody Conjugation Kit (Bio-Rad). For the conjugation reactions both RPH-120 and control IgG1 were used in concentration 1.0 mg/ml. Cryo-sections from human and cynomolgus monkey tissues were prepared. The study was designed to meet the requirements and to follow guidelines of FDA and EMA: “Points to Consider in the Manufacture and Testing of Monoclonal Antibody Products for Human Use” (Miele and Stein, 1997) and “Guideline on Development, Production, Characterization and Specifications for Monoclonal Antibodies and Related Products” (European Medicines Agency, 2016). The assessment of tissue viability indicated that the panel of human and Cynomolgus monkey tissues were viable. The method development using atezolizumab was carried out to confirm specificity of staining in the test tissues using both RPH-120-AP and Atezolizumab-AP using IgG1-AP as a negative control.

Mixed lymphocyte reaction (MLR) assay. Bioactivity of RPH-120 was assessed using a MLR assay to evaluate and compare the potencies of RPH-120, atezolizumab and pembrolizumab in T cell activation experiment. The T cell activation was quantified by measuring concentration of interleukin-2 (IL-2) secreted by T cells. Dendritic cells (DC) and CD4^+^ T cells were isolated from human peripheral blood mononuclear cells (PBMC). CD4^+^ T cells and dendritic cells were seeded into the 96-well plate and treated with the testing antibodies: pembrolizumab and RPH-120 in Plate1, pembrolizumab and atezolizumab in Plate 2. Half-maximal effective concentration (EC50) values were derived from raw dose-response data using non-linear four-parameter regression fit algorithm by GraphPad Prism software package.

*In vivo* efficacy of RPH-120 on growth of human lung adenocarcinoma HCC-827 subcutaneous xenografts in CD34^+^ humanized mice. Human lung adenocarcinoma HCC-827 cells provided by The Cell Bank of Chinese Academy of Sciences (Shanghai, China) were used in this study. The cells were sub-cultured within 10 passages before inoculated into mice. The mycoplasma-free cells were kept on ice before inoculation. Total of 5.0E + 06 cells resuspended in 0.2 ml PBS + Matrigel mixed with 1:1 volume ratio were injected subcutaneously to each of the 78 mice selected from a pool of 100 mice (hCD45 > 15%), based on hCD45/hCD3/hCD4 data for the pre-selection.

When the average tumor volume reached 50–80 mm^3^, 50 tumor bearing mice were selected and randomized into five groups (10 mice per group) according to the tumor volume and hCD45^+^rate. Dosing of test antibodies got started on the same day after animal randomization. The groups are indicated below (Table 1). The day of grouping and dosing was denoted as Day 0. The drug was administered intravenously in regimen two times a week during 6 weeks.

**TABLE 1:** Grouping and dosing regimen in the *in vivo* efficacy experiment.

Groups		Concentration mg/mL	Dosage	
			mL/kg	mg/kg
1	Vehicle	N/A	10	N/A
2	Atezolizumab	1	10	10
3	RPH-120 low dose	2	10	20
4	RPH-120 middle dose	8	10	80
5	RPH-120 high dose	20	10	200

Measurement: Body weight and tumor volume were measured twice a week during 6 weeks after grouping. Tumor volume: the tumor volume (V) was calculated as follows: V = (length × width2) /2. The individual relative tumor volume (RTV) was calculated as follows: RTV = V_t_/V_0_, where V_t_ was the volume on each day, and V_0_ was the volume at the beginning of the treatment. TGI: The tumor growth inhibition (TGI) was calculated as follows: TGI% = (1-T/C)×100%, T and C as the mean TVs of the treatment and control groups on the measurement day. Results were demonstrated as mean ± S.E.M. Comparisons were made by Dunnett’s multi-comparison test, *p* < 0.05 was considered significant.

In a single-dose toxicity, toxicokinetic and dose-range finding study, 12 male and 12 female cynomolgus monkeys (*Macaca fascicularis*) received i. v. RPH-120 by either bolus or 1-h infusion as specified in (Table 2). After dosing, animals were observed for 8-weeks to assess the reversibility, persistence, or delayed occurrence of effects.

**TABLE 2:** Dose range finding and TK study design by RPH-120 treatment.

Group	No. of Animals		Dose level (mg/kg)	Dose concentration (mg/ml)	Dose volume (ml/kg)
	Male	Female			
1 (Control, IV Injection)	2	2	0	0	2
2 (Low, IV Injection)	2	2	12.5	6.25	2
3 (Mid, IV Injection)	2	2	50	25	2
4 (High, IV Injection)	2	2	200	40	5
5 (Low, Infusion)	2	2	12.5	2.5	5
6 (High, Infusion)	2	2	200	40	5

Animal care and use were conducted in an AAALAC-accredited facility in adherence with applicable animal welfare.

Clinical symptoms, body weight and food consumption were evaluated, blood samples were collected for pharmacokinetic RPH-120 analysis and cytokine analysis (IL-2, IL-6, IFNγ). Laboratory safety evaluations included clinical and biochemical blood analysis, histology coagulation profile, urinalysis and microscopic examination of urine sediment. Local tolerance was assessed using a modified Draize technique.

Blood samples for bioanalytical and toxicokinetic analysis were collected *via* femoral vein on Days 1, 2, 4, 6, 8, 10, 14, 21, 28, 35, 42, 49, and 56 of the dosing phase. Samples were collected predose and approximately 0.25, 0.5, 3, 6, and 24 h postdose for Groups 1, 2, 3, and 4 on the day of dosing. Samples also were collected predose and approximately 0.5, 1, 3, 6, and 24 h postdose for Groups 5 and 6 on the day of dosing. Animals were not fasted for sample collections, unless fasted because of other study procedures. The time was counted from the start of the dosing of test compounds.

The toxicokinetic analysis of the serum samples was performed using specifically validated method. It included parameters listed in the following: C_0_: concentration at zero time (IV Injection Groups), C_max_: Peak concentration, T_max_: Time to peak concentration, AUC_0-t_: Area under the concentration-time curve calculated using linear trapezoidal method (linear interpolation). Phoenix WinNonlin was used for the data analysis. Statistical analysis of the results was conducted based on corresponding predose and control group data. Female and male data of each group was set together and statistically analyzed.

## Results

RPH-120 is a fully human anti-PD-L1 IgG1 class monoclonal antibody with grafted scFv (Single Chain Fragment Variable). The entire extracellular fragment of hPD-L1, (Uniprot database, Q9NZQ7), residues 19–238, was genetically fused to mouse IgG-Fc fragment (Uniprot database, P01868), residues 90–324, and used for antibody selection process. Since PD-L1 amino acid residues participating in binding PD-1 were mapped within the region D26 – R125, it can be assumed that the epitope recognized by RPH-120 could be located within the specified region or its vicinity. RPH-120 was stable in its formulation buffer for at least 3 years after manufacturing and its other critical quality attributes, including purity, integrity, and biological activity remain within specifications (data not shown).

### Binding Studies

**FIGURE 1:**
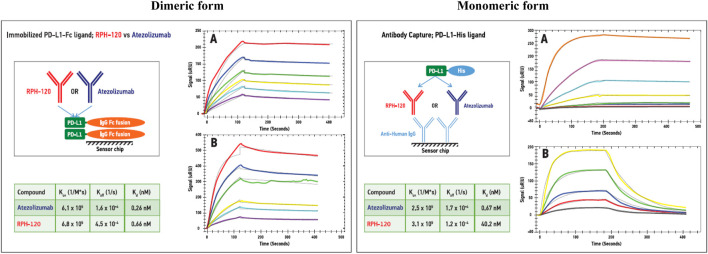
SPR analysis of the RPH-120 or atezolizumab binding parameters to dimeric or monomeric forms of human PD-L1: Schematic presentation of alternative capturing approaches. Dimeric form: PD-L1-Fc fusion protein was immobilized on a CM5 sensor chip, then series of SPR sensograms of binding of either RPH-120 or atezolizumab at various concentrations, were generated. Monomeric form: Anti-human capturing antibodies were immobilized on a CM5 sensor chip, then RPH-120 or atezolizumab were loaded on the capturing antibodies followed by various concentrations of monomeric (PD-L1-His) tagged protein. Series of sensograms of binding of the monomeric form were generated and analysed.Panels **A** – Aatezolizumab; Panels **B** – RPH-120.

Surface plasmon resonance experiments were carried out to compare binding properties of RPH-120 and atezolizumab toward monomeric and dimeric forms of PD-L1: recombinant human His-PD-L1 and recombinant human PD-L1-Fc fusion protein, respectively. Both ligands were tested by SE-HPLC that confirmed monomeric state of the former protein and dimeric state of the latter one (see Supplementary Figure S1). The experimental data showed that binding affinities of immobilized dimeric PD-L1-Fc for RPH-120 and atezolizumab were similar, KD values being 0.66 and 0.26 nM, respectively (Figure 1, Dimeric form). Binding affinity of monomeric His-tagged PD-L1 for the comparator atezolizumab was about 2-log higher than for RPH-120, 0.67 vs. 40.2 nM, respectively (Figure 1, Monomeric form). When both monomeric and dimeric PD-L1 forms interacted with immobilized RPH-120 or atezolizumab, KD of RPH-120 interaction with monomeric and dimeric PD-L1 were 14.3 and 0.45 nM respectively, whereas for the atezolizumab the values were not significantly different: 0.62 and 0.19 nM, respectively. The lower affinity of RPH-120-PD-L1-His interaction is explained by higher rate of dissociation, whereas association phases for RPH-120 and atezolizumab look almost identical. Thus, RPH-120 binds to the dimeric PD-L1 with 2-log higher affinity than to the monomeric form and this finding may indicate greater selectivity of this molecule toward the dimeric form compared to atezolizumab.

**FIGURE 2:**
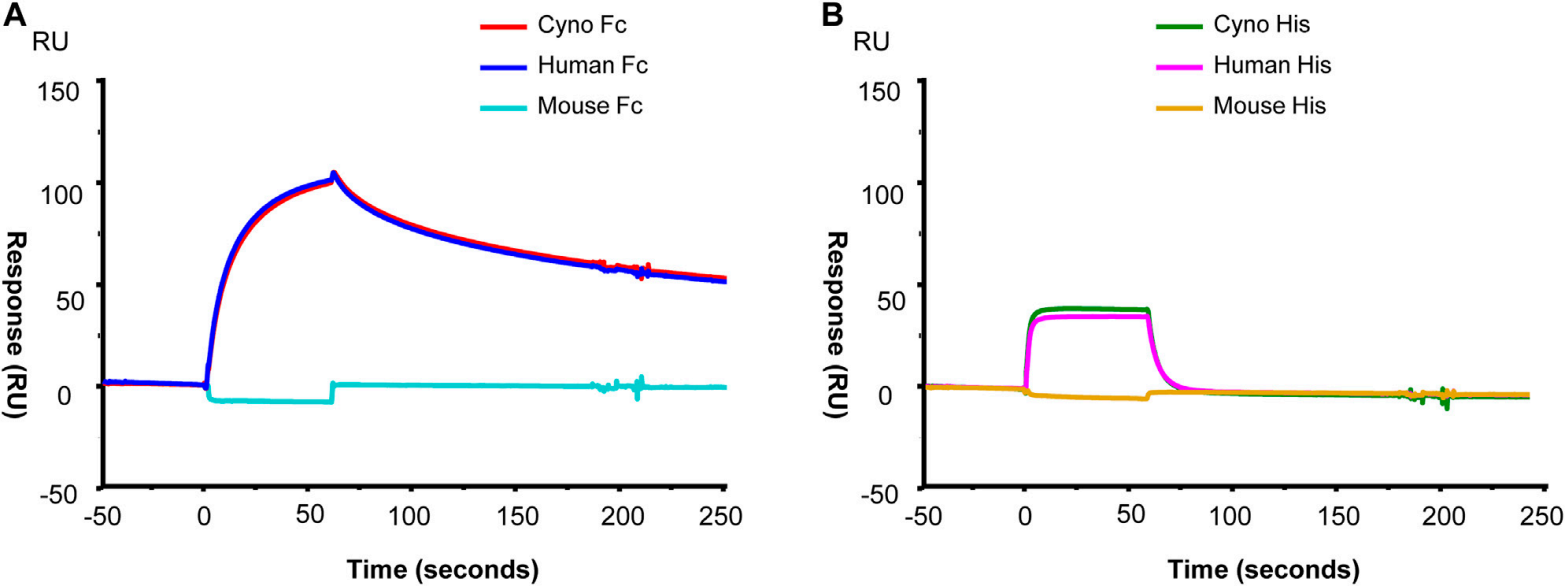
SPR analysis of the RPH-120 binding to human, cynomolgus or mouse PD-L1. RPH-120 was immobilized on a CM5 sensor chip then human, monkey or mouse Fc-tagged PD-L1 (Panel **A**) or His-tagged PD-L1 (Panel **B**) proteins were applied at 30 µl/min for 1 min to generate the sensograms.

RPH-120 binding to PD-L1 from different species. Binding properties of RPH-120 towards PD-L1 from different species was also analyzed using surface plasmon resonance. Three different species mouse, Cynomolgus monkey and human were tested. It was demonstrated that RPH-120 efficiently binds both human and cynomolgus PD-L1, however apparently does not bind mouse PD-L1 (Figure 2). In contrast to this finding, atezolizumab has shown to bind PD-L1 from all three species including murine, with comparable affinities (Leighton et al., 2016).

### Immunohistochemistry of RPH-120 in Normal Human Tissues

**FIGURE 3:**
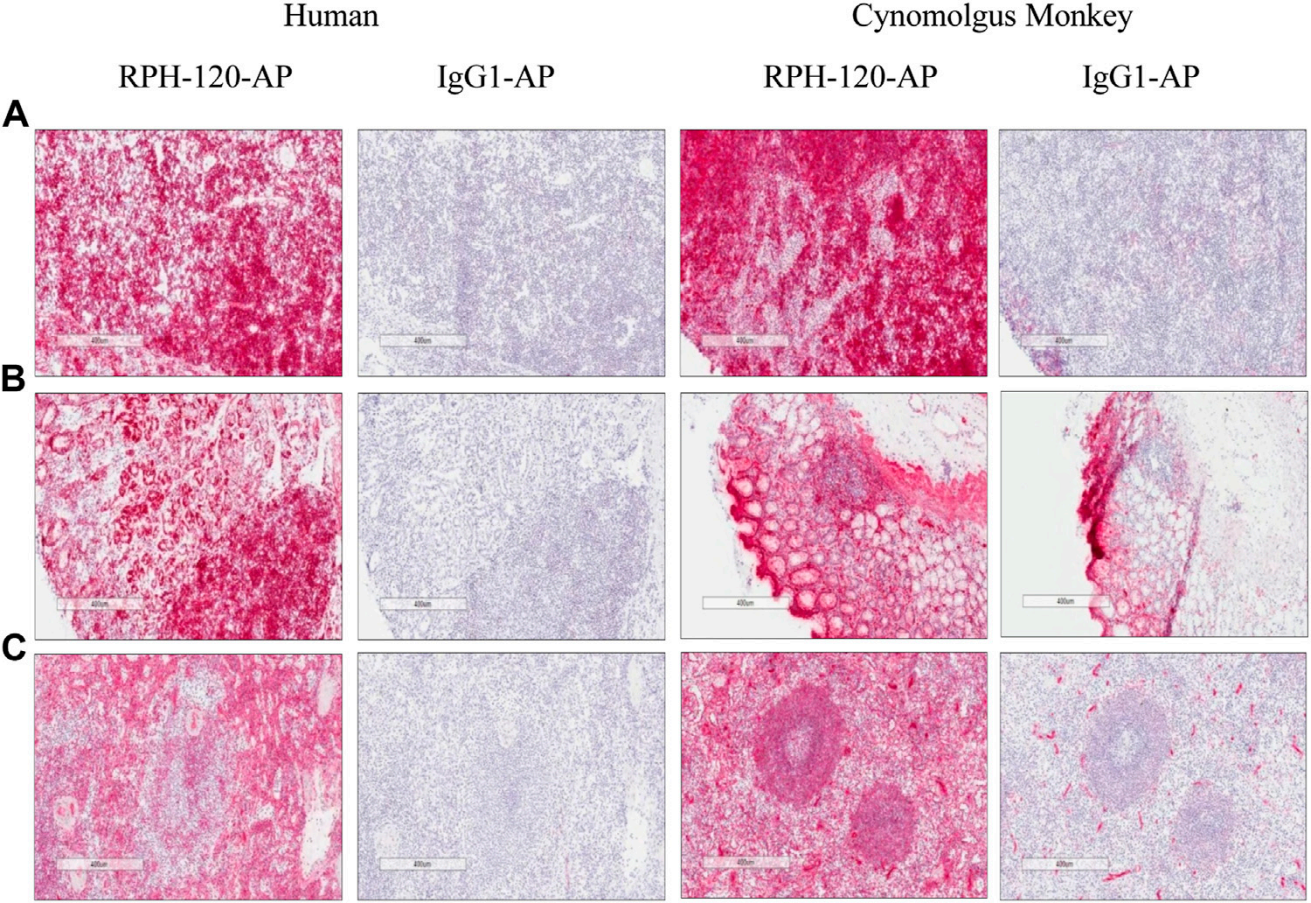
IHC detection of endogenous PD-L1 by RPH-120 in several tissues. RPH-120 or control human IgG1 were conjugated to Alkaline Phosphatase (AP) and used for IHC studies. There are three groups of tissues from human and Cynomolgus monkey are shown here: Panel **A**: Lymph Node, Panel **B** Gastric Body tissue and Panel **C** - spleen.

The experiment showed that specific positive membrane staining with RPH-120-AP was observed in lymphocytes and epithelial cells in a number of tissues examined as well as in hepatocytes, alveolar macrophages and ependymal cells. Diffuse specific staining was observed in the neuropil. As an example, several staining images of lymph nodes, gastric body and spleen are shown in Figure 3. The membrane and diffuse staining observed is consistent with PD-L1 distribution as described in the literature (Francisco et al., 2010; Chen et al., 2017) and is therefore considered to be target related. Specific positive cytoplasmic staining with RPH-120-AP was also observed in fibrovascular stroma and smooth muscle in the majority of tissues examined in addition to a number of other cell types. This staining is considered to be specific for RPH-120-AP, however the cytoplasm is considered not accessible to biotherapeutics *in vivo* (Leach et al., 2010). Generally, the tissue cross-reactivity study showed good comparability of human and Cynomolgus monkey tissue staining.

**FIGURE 4:**
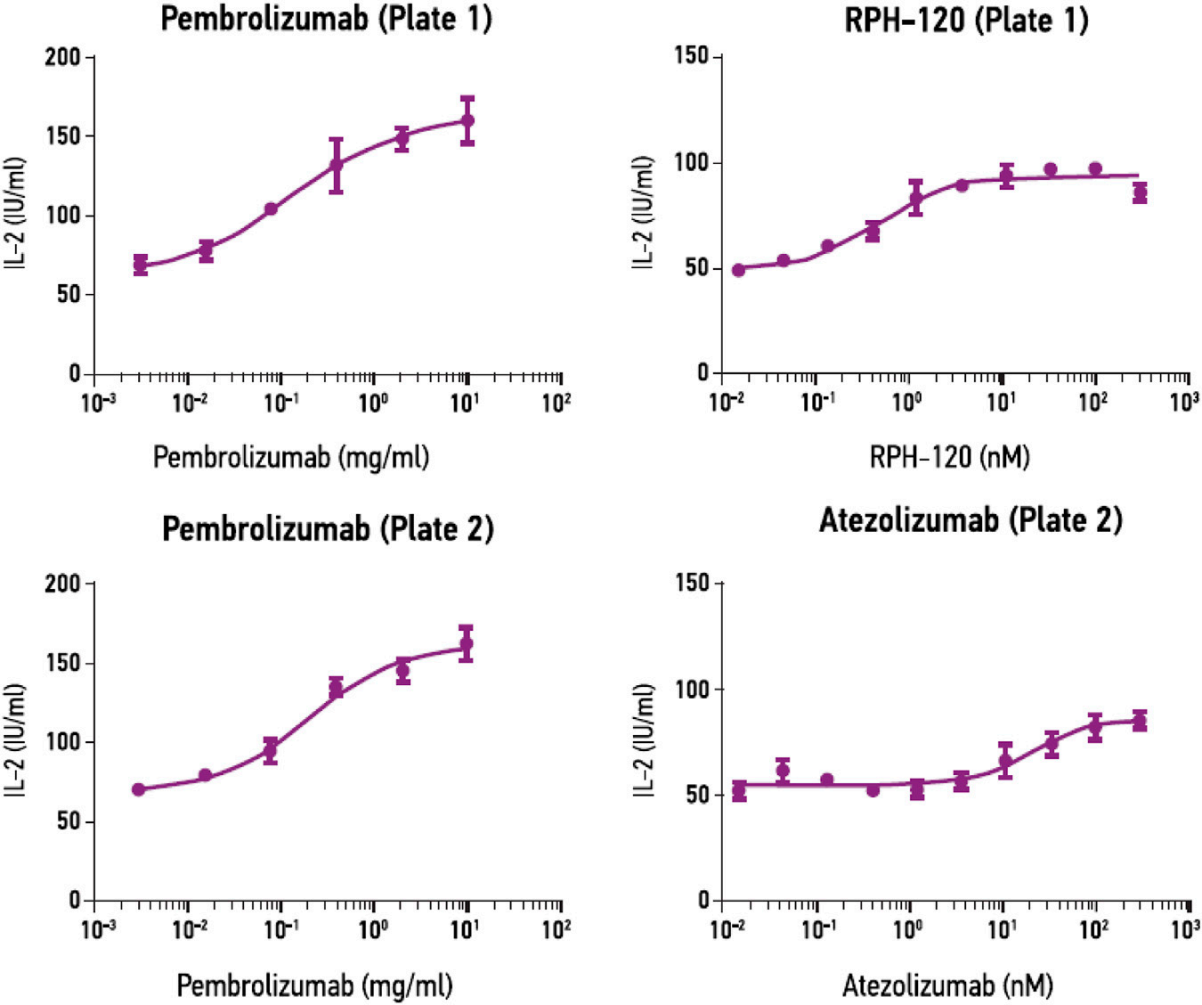
RPH-120 enhances CD4^+^ T cell activation in MLR. RPH-120 and atezolizumab dose-response curve of IL-2 secretion were monotonous and the experimental points were fit into a curve using non-linear four-parameter regression algorithm to determine EC50 values.

Mixed lymphocyte reaction (MLR) assay. MLR assay was used to estimate bioactivity of RPH-120 by its capability to increase secretion of IL-2 by activated T lymphocytes. A PD-L1 inhibitor atezolizumab and PD-1 inhibitor pembrolizumab were used as comparators. EC50 values for RPH-120, atezolizumab and pembrolizumab were 0.47, 21.53 and 0.87 nM (1.23 nM for Plate 2) respectively (Figure 4). The results of the MLR assay demonstrated that biological activity of the RPH-120 was 40-fold higher than that of atezolizumab. T cell activation by the internal control (pembrolizumab) was consistent with historical data, thereby verifying data obtained.

**FIGURE 5:**
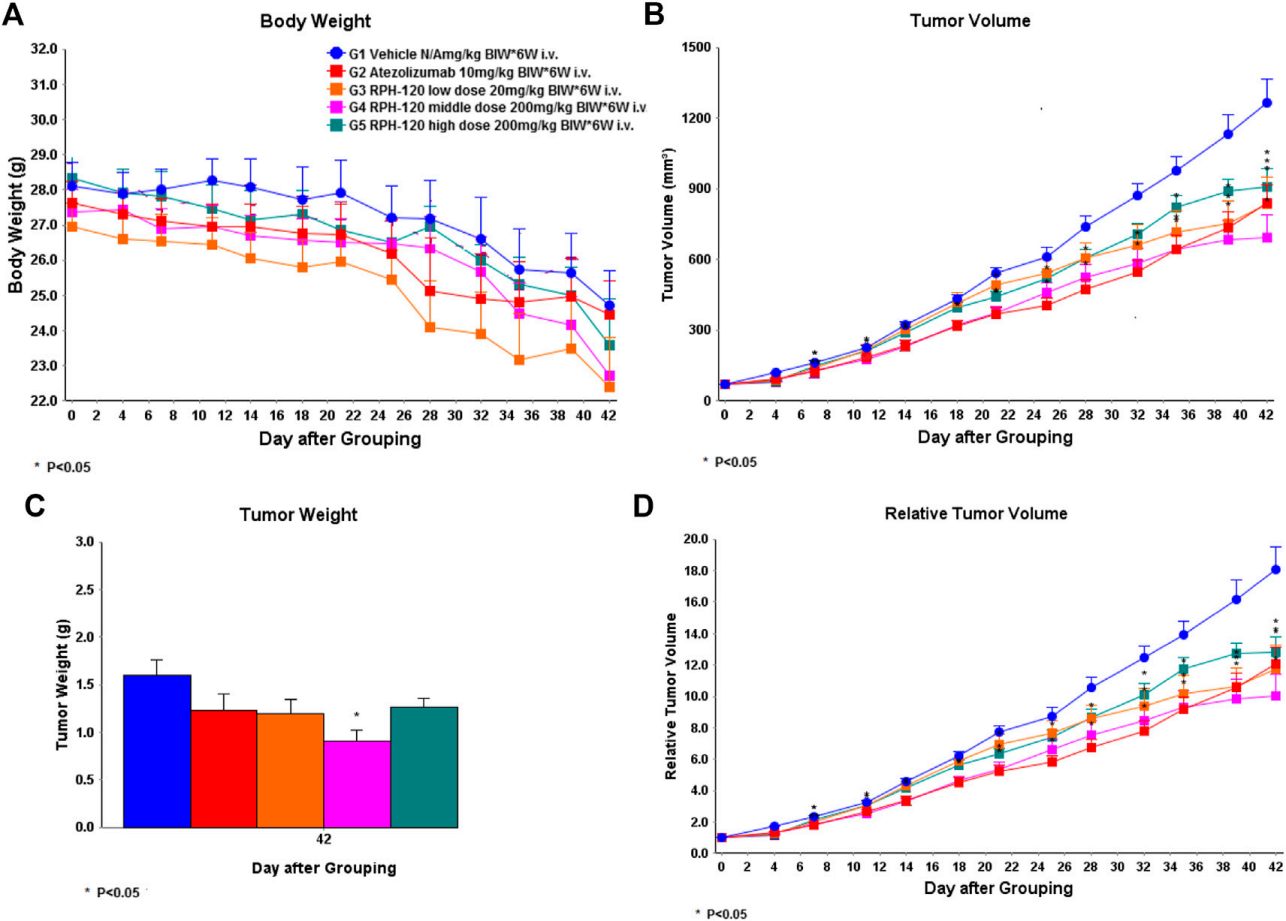
The effect of RPH-120 on growth of human lung adenocarcinoma HCC-827 subcutaneous xenografts in CD34^+^ humanized Mice. Panel **A** - Body Weight in the indicated mouse groups; Panel **B** - Tumor Volume (V) in the indicated mouse groups. It was calculated as follows: V = V = (length ×width2) / 2. Panel **C** - Tumor Weight was measured on Day 42. Panel **D** - Relative tumor volume (RTV) was calculated as follows: RTV = V_t_/V_0_, where V_t_ was the volume on each day, and V_0_ was the volume at the beginning of the treatment.

Inhibition of growth of HCC-827 xenografts *in vivo.* Anti-cancer efficiency of RPH-120 *in vivo* was analyzed using human lung adenocarcinoma xenograft model in CD34^+^ humanized mice. Human lung cancer cells HCC-827 subcutaneous xenografts grew well in vehicle treatment group 1 (Figure 5), demonstrating that the HCC-827 xenograft model was successfully established in hCD34+ mice in this study. An obvious weight loss was observed after 4 weeks of treatment with both RPH-120 (20, 80, and 200 mg/kg) and atezolizumab (10 mg/kg), and for about 30% of animals the treatment was stopped. The average tumor volume in Vehicle group 1 reached about 1,264.86 mm^3^ on the 42nd day. Treatment with RPH-120 at 80 mg/kg showed tumor growth inhibition, that was demonstrated by a statistically significant decrease in both tumor volume, relative tumor volume and tumor weight throughout the entire study compared to Vehicle group 1 (*p*-value < 0.01). Treatment with RPH-120 at low dose 20 mg/kg significantly decreased tumor volume only on day 4 and day 35–42, and RPH-120 at high dose of 200 mg/kg reduced tumor volume only on day 4, day 21, day 32, and day 42. Tumor growth inhibition (TGI) by low dose group reached 34% and TGI by high dose group reached 28% on the last day 42. Treatment with anti-PD-L1 antibody atezolizumab inhibited tumor growth which was assessed by tumor volume and relative tumor volume compared to Vehicle group 1. Tumor growth inhibition of 34% by atezolizumab was observed on day 42. These data demonstrated that RPH-120 possesses anti-cancer effect on lung adenocarcinoma xenografts in humanized mice.

#### Single Dose Toxicity, Toxicokinetic and Dose Range Finding Study

Administration of RPH-120 to Cynomolgus monkeys intravenously by bolus injection at all three doses 12.5, 50, 200 mg/kg or 1-h infusion at 12.5 and 200 mg/kg were well tolerated with no effects on body weight, food consumption or clinical observations. There was no mortality and no local irritation noted by administration of RPH-120. Also, we didn’t observe abnormalities in hematology, clinical chemistry, coagulation or urinalysis tests for animals of either control or any RPH-120 treated groups. Cytokine analysis showed that no remarkable changes were observed in IL-2, IL-6, and IFNγ levels upon bolus injection and 1-h infusion of RPH-120.

**FIGURE 6:**
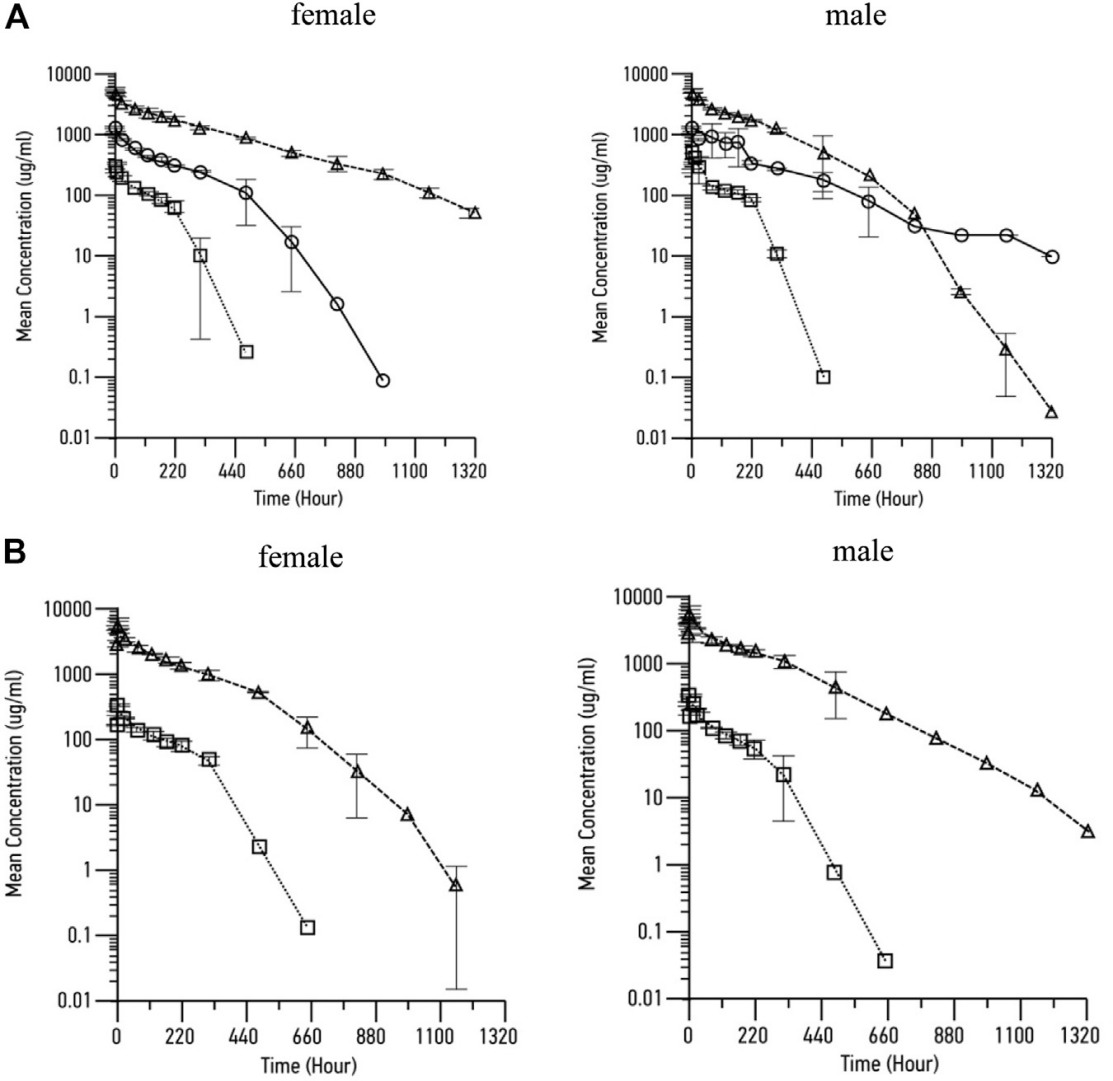
Pharmacokinetic profile of RPH-120 in male and female Cynomolgus monkeys. Panel **A** – mean RPH-120 concentrations in sera of female and male monkeys after single dose i.v. bolus injection of RPH-120 (doses 12.5; 50 and 200 mg/kg); Panel **B** - mean RPH-120 concentrations in sera of female and male monkeys after single dose i.v. infusion of RPH-120 (doses 12.5 and 200 mg/kg).

Following both bolus injection and infusion administration of RPH-120, increase in maximum concentration and mean exposure was noted (Table 3 *,* Figure 6). Mean concentration–time profiles for serum RPH-120 were qualitatively similar for males and females at dose range from 12.5 to 200 mg/kg, indicating no gender difference for RPH-120 concentration in monkey serum. The increases in C_max_ and AUC_0-t_ values were generally dose proportional except the AUC_0-t_ value of RPH-120 in female monkey at 200 mg/kg dose level was greater than dose proportional. For 1 h infusion groups, the increases in C_max_ and AUC_0-t_ values were generally dose proportional. After intravenous bolus injection of RPH-120 the mean time of peak concentration (T_max)_ values ranging from 0.25 to 1.75 h. Thus, RPH-120 has an acceptable toxicokinetic profile and was well tolerated by all animals when administered up to 200 mg/kg.

**TABLE 3:** Summary of toxicokinetic data in monkey serum after RPH-120 administration.

RPH-120 type of administration	Dose level, mg/kg	Male				Female			
		C_max,_ µg/ml	AUC_0-t,_ Hour*µg/ml	Tmax, h	T1/2, h	C_max,_ µg/ml	AUC_0-t,_ Hour*µg/ml	Tmax, h	T1/2, h
i.v. injection	12.5	553	41,300	0.25	37.2	306	31,400	0.38	33.4
i.v. injection	50	1,360	282,000	1.75	128	1,370	189,000	0.38	45.6
i.v. injection	200	6,050	963,000	1.75	53.8	5,500	1,170,000	0.25	156
1 h infusion	12.5	332	28,400	1	54.6	341	40,000	1	38.2
1 h infusion	200	6,130	838,000	1	96.7	6,130	843,000	1	60.4

## Discussion

Inhibitory T cell signaling modulates immune homeostasis but may restrict immune responses against cancer. One of the costimulatory ligand, PD-L1, was identified as a negative regulator of antitumor T cell-mediated immunity that acts by binding to and activation of its receptor PD-1. It was demonstrated that PD-L1 is upregulated in many different types of tumors and in tumor microenvironment. Thus, inhibiting the interaction of PD-L1 and PD-1 is an important rationale for antibody blockade of immune checkpoint PD-L1 in order to restore tumor-specific T cell immunity against tumor cells. It allows to “release the brakes” and enable attack of cancer cells by T-lymphocytes. Several antibodies blocking the interaction of PD-1 with PD-L1 have shown the ability to enhance antitumor immune responses in diverse cancers (Wang et al., 2014; Stewart et al., 2015). Currently nivolumab, pembrolizumab, and cemiplimab are registered PD-1 inhibitors and atezolizumab, durvalumab and avelumab are registered PD-L1 inhibitors. These agents are now a standard of care in various malignancies, such as melanoma, non-small cell lung cancer, squamous head and neck cancer, microsatellite-unstable colorectal cancer, and other types of cancers (Sun et al., 2020). PD-1 and PD-L1 inhibitors have some specific features and differences in efficacy and safety profiles (Pillai et al., 2018; Spagnuolo and Gridelli, 2018).

RPH-120 is a fully human IgG1 antibody (with) specifically targeting to PD-L1. Its preclinical development was done in accordance with the ICH international guidelines: ICH S6, ICH S9. RPH-120 (S6 International Conference on Harmonisation, 1997; S9 International Conference on Harmonisation, 2009), similar to other IgG1 antibodies, consists of two heavy (449 amino acids each) and two light (214 amino acids) chains. In the heavy chain amino acid residue 300 (asparagine) was substituted to alanine (Asn300Ala) that causes an abolishment of the glycosylation and, therefore, a low affinity to Fcγ receptors confirmed by experiments *in vitro*. Atezolizumab also has such a substitution at position 298 which leads to the same consequences, a low Fc-mediated activity. Atezolizumab was used in our preclinical study program as a positive control, although atezolizumab is a humanized murine antibody and RPH-120 is a fully human antibody.

RPH-120 showed considerable difference in binding to dimeric and monomeric forms of PD-L1. Indeed, binding affinity to dimers of PD-L1 was much higher than to monomers, Kd were 0.43 and 14.3 respectively, whereas for atezolizumab binding Kd for monomers and dimers of PD-L1 were 0.62 and 0.19 respectively. Additional Biacore study also demonstrated that RPH-120 can bind to human and monkey PD-L1, but not to mouse PD-L1. This important RPH-120 feature (as compared to atezolizumab) imposed specific restrictions to selection of appropriate *in vivo* model in preclinical testing. We concluded that RPH-120 is highly selective for dimeric form of PD-L1 in humans and non-human primates, whereas do not exert any meaningful blockade of PD-L1 monomers. Moreover, binding of RPH-120 to PD-L1 dimers is similar to atezolizumab’s binding. Since several publications postulated that dimers have more prominent functional activity than monomers (Chen et al., 2010), we suggested that RPH-120 might be capable of producing anticancer activity comparable to that of other PD-L1 inhibitors. Immunohistochemical studies evaluated RPH-120 reactivity to a panel of normal tissues and assessed nontarget tissue binding. The observed staining results are consistent with PD-L1 distribution as described in the literature and showed good comparability of human and Cynomolgus monkey tissue staining.

Anticancer efficacy of RPH-120 was studied using *in vitro* and *in vivo* models. *In vitro* activity was analyzed using MLR that relies on assessment of IL-2 production by activated lymphocytes isolated from PBMC. Activity of RPH-120 was compared to another two immune checkpoint inhibitors, PD-L1 inhibitor atezolizumab and PD-1 inhibitor pembrolizumab. RPH-120 demonstrated ability to activate T-lymphocytes in lower concentration than atezolizumab, but comparable to pembrolizumab. Efficacy of RPH-120 *in vivo* was further tested using human lung adenocarcinoma HCC-827 cells xenografts in CD34^+^ humanized mice. Since binding experiments showed that RPH-120 binds to human and monkey PD-L1, but does not cross-react with mouse PD-L1, syngeneic *in vivo* models could not be used for estimation of drug’s efficacy and therefore, we used CD34^+^ humanized mice. RPH-120 at dose 80 mg/kg demonstrated statistically significant tumor growth inhibition. Thus, RPH-120 produced promising *in vitro* and *in vivo* anticancer activity in described models.

A single-dose toxicity, toxicokinetic and dose range finding study in cynomolgus monkeys provided a RPH-120 PK profile that can support clinical testing. We used either in several doses and then animals were observed for 8-weeks to assess the effects. Mean concentration–time profiles for serum RPH-120 were qualitatively similar for both genders in monkey serum. Generally, RPH-120 was well tolerated, when administered to cynomolgus monkeys as i.v. bolus or 1-h infusion in doses 12.5 mg/kg – 200 mg/kg, with no adverse effects on any parameters. There were no RPH-120-related clinical signs of local or systemic toxicity up to 200 mg/kg dose observed during the study.

In conclusion, RPH-120 demonstrated promising *in vitro* and *in vivo* activities with acceptable toxicokinetic and safety profiles. Collectively, RPH-120 is considered a potent anti-PD-L1 drug candidate for cancer immunotherapy.

## Data Availability

The raw data supporting the conclusions of this article will be made available by the authors, without undue reservation.
