# Supplementary material for: Preclinical Characterization of a Novel Anti-Cancer PD-L1 Inhibitor RPH-120
Source: Front Pharmacol. 2021 Aug 11;12:723038. doi: 10.3389/fphar.2021.723038 (PMC8386121; doi:10.3389/fphar.2021.723038)
Supplement: Supplementary file 1 [file Presentation1.pptx]

## Slide 1
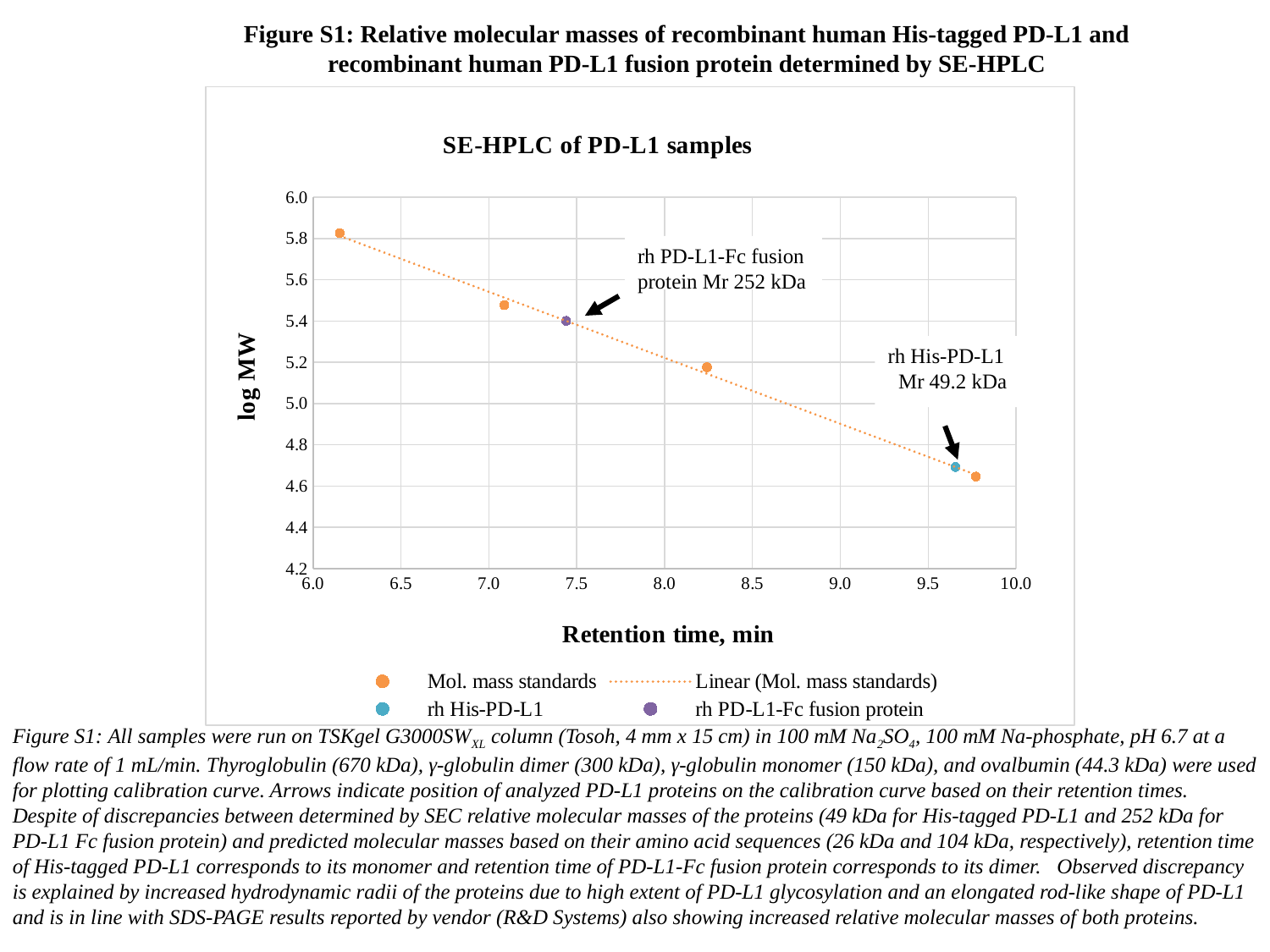

# Figure S1: Relative molecular masses of recombinant human His-tagged PD-L1 and recombinant human PD-L1 fusion protein determined by SE-HPLC
### Chart: SE-HPLC of PD-L1 samples
| Category | Mol. mass standards | rh His-PD-L1 | rh PD-L1-Fc fusion protein |
|---|---|---|---|rh PD-L1-Fc fusion protein Mr 252 kDa
rh His-PD-L1 Mr 49.2 kDa
Figure S1: All samples were run on TSKgel G3000SWXL column (Tosoh, 4 mm x 15 cm) in 100 mM Na2SO4, 100 mM Na-phosphate, pH 6.7 at a flow rate of 1 mL/min. Thyroglobulin (670 kDa), γ-globulin dimer (300 kDa), γ-globulin monomer (150 kDa), and ovalbumin (44.3 kDa) were used for plotting calibration curve. Arrows indicate position of analyzed PD-L1 proteins on the calibration curve based on their retention times.
Despite of discrepancies between determined by SEC relative molecular masses of the proteins (49 kDa for His-tagged PD-L1 and 252 kDa for PD-L1 Fc fusion protein) and predicted molecular masses based on their amino acid sequences (26 kDa and 104 kDa, respectively), retention time of His-tagged PD-L1 corresponds to its monomer and retention time of PD-L1-Fc fusion protein corresponds to its dimer. Observed discrepancy is explained by increased hydrodynamic radii of the proteins due to high extent of PD-L1 glycosylation and an elongated rod-like shape of PD-L1 and is in line with SDS-PAGE results reported by vendor (R&D Systems) also showing increased relative molecular masses of both proteins.
